# Pediatric T-cell prolymphocytic leukemia with an isolated 12(p13) deletion and aberrant CD117 expression

**DOI:** 10.1186/2162-3619-1-7

**Published:** 2012-04-18

**Authors:** Michael Bellone, Annika M Svensson, Ann-Leslie Zaslav, Silvia Spitzer, Marc Golightly, Mahmut Celiker, Youjun Hu, Yupo Ma, Tahmeena Ahmed

**Affiliations:** 1 Department of Pathology, Stony Brook University Medical Center, Stony Brook, NY 11794, USA; 2 Cytogenetics, Department of Pathology, Stony Brook University Medical Center, Stony Brook, NY 11794, USA; 3 Molecular Genetics, Department of Pathology, Stony Brook University Medical Center, Stony Brook, NY 11794, USA; 4 Flow Cytometry, Department of Pathology, Stony Brook University Medical Center, Stony Brook, NY 11794, USA; 5 Department of Pediatrics, Stony Brook University Medical Center, Stony Brook, NY 11794, USA; 6 Stony Brook University Medical Center, UH Level 2, Rm, 766, Stony Brook, NY 11794-7300, USA

**Keywords:** T-cell Prolymphocytic Leukemia, Pediatric T-cell Lymphomas, Alemtuzumab, TCR rearrangement, CD117, 12p13

## Abstract

T-cell Prolymphocytic leukemia (T-PLL) is a rare post-thymic T-cell malignancy that follows an aggressive clinical course. The classical presentation includes an elevated white blood cell (WBC) count with anemia and thrombocytopenia, hepatosplenomegaly, and lymphadenopathy. T-PLL is a disease of the elderly and to our knowledge it has never been described in the pediatric age group. We report a case of T-PLL in a 9 year old male who was initially diagnosed with T-cell acute lymphoblastic lymphoma (ALL), the diagnosis was later refined to T-PLL following additional analysis of bone marrow morphology and immunophenotype. Two unusual findings in our patient included CD117 expression and an isolated chromosomal 12(p13) deletion. The patient failed to respond to standard ALL induction chemotherapy, but achieved complete remission following treatment with a fludarabine and alemtuzumab-based regimen.

## Introduction

T-cell prolymphocytic leukemia (T-PLL) is an aggressive lymphoproliferative disorder that represents approximately 2% of all mature lymphocytic leukemias in adults. Most patients present with hepatosplenomegaly, lymphadenopathy, and marked lymphocytosis. Less commonly skin lesions and serous effusions develop. T-PLL is characterized by proliferation of small to medium-sized prolymphocytes with nongranular basophilic cytoplasm; round, oval, or markedly irregular nuclei; and a prominent nucleolus. In approximately 20% of cases a "small cell variant" is seen. The immunophenotype of T-PLL cells resembles that of a mature post-thymic T-cell with expression of CD2, CD3, and CD7. The T-cell receptor (TCR) beta/gamma genes are clonally rearranged. The most frequent chromosomal abnormalities in T-PLL include inversion of chromosome 14 with breakpoints in the long arm of q11 and q32 and abnormalities of chromosome 8 [1].

T-PLL arises sporadically in adults and is mainly a disease of the elderly with a median age at onset of 65 years [2]. A mature T-cell malignancy with phenotypic and genotypic features indistinguishable from T-PLL has been described in patients with ataxia-telangiectasia [3]. In contrast to sporadic cases T-PLL, this entity is seen in younger adults with a median age of onset of 31 years.

We present a case of a 9-year-old male diagnosed with T-PLL based on morphology, immunophenotype, cytogenetic and molecular T-cell receptor studies of bone marrow. A thorough literature search through Medline, Pubmed, and Google scholar did not reveal any previous description of T-PLL presenting in the pediatric age group. Because this patient showed no clinical or cytogenetic features of ataxia-telangiectasia, this is likely a case of sporadic T-PLL, making it even more intriguing.

## Case presentation

The patient is a 9-year-old African-American male with no significant personal or family medical history who was well until about 2 months ago when he started having intermittent fevers, nonbloody nonbilious vomiting, fatigue and abdominal pain. He completed 2 courses of antibiotics during that time. Three days prior to admission, he went back to his pediatrician who started him on another course of antibiotics. A CBC was done at that time which revealed severe anemia. So he was sent to the local community hospital emergency department. Physical examination revealed fever (temperature of 38.9°C), periorbital edema, bilateral pitting pedal edema, hepatomegaly (5 cm), splenomegaly (3 cm), and cervical and axillary lymphadenopathy. A complete blood count showed a hemoglobin of 4.4 g/dl, a white blood cell count of 10.6 × 10^3^/μl with 98% lymphocytes and 2% blasts, and a platelet count of 119 × 10^3^/μl. Blood biochemical analysis revealed elevated LDH of 739 units/L (reference range 94-250). Additional studies revealed hypoalbuminemia (2.5 g/dl, reference range 3.5-4.8) and increased hyperferritinemia (1,510 ng/mL, reference range 22.0-322.0). Serology for HTLV-1 was negative while CMV IgG was positive suggesting prior exposure. Bone marrow aspiration was interpreted as T-ALL based on morphology and cell surface marker expression as detailed below.

Four-drug induction chemotherapy following COG protocol AALL0434, consisting of intrathecal cytarabine and systemic vincristine, prednisone, daunorubicin, and PEG-asparaginase was started. Day 8 bone marrow aspirate showed decrease in leukemic cells. Induction chemotherapy was continued as planned. Bone marrow aspiration done on day 15 of induction showed progressive disease. The lack of response to standard T-ALL induction chemotherapy prompted a revision of the diagnosis. Evaluation of the bone marrow specimens revealed prolymphocytic appearing cells that lacked expression of markers of immaturity such as CD34, TdT, and CD1a even though there was expression of CD117. This unusual immunophenotype, morphology and lack of clinical response, resulted in a revision of the diagnosis to T-cell prolymphocytic leukemia.

Following change in diagnosis and progressive disease under standard induction chemotherapy, induction chemotherapy was abandoned and the patient was started on nelarabine as a single agent with intention to add cyclophosphamide and cytarabine. Lack of evidence of tumor lysis at the end of nelarabine therapy prompted a repeat of bone marrow aspiration which showed persistent disease. The child then was treated with another regimen termed FLACC, which consisted of fludarabine, cytarabine, cyclophosphamide, and alemtuzumab with filgastrim (G-CSF) support and was described in detail by Williams et al. [4], who used this regimen to successfully treat a child with refractory T-cell post-transplant lymphoproliferative disorder (PTLD). The patient received valacyclovir for prophylaxis against CMV reactivation and pentamidine for PCP prophylaxis.

Upon hematopoietic recovery, bone marrow aspiration and biopsy showed trilineage maturation with no evidence of leukemia by morphology or flow cytometry confirming remission status. The child had no biological siblings as potential stem cell donor. While the search for a suitable stem cell source was underway, the child received a second course of the FLACC therapy to sustain remission status. During neutropenic phase of this second cycle the child developed respiratory distress with fevers. At the time of neutrophil recovery, respiratory distress worsened necessitating mechanical ventilation. This was followed by failure of renal and hepatic systems. All blood cultures remained negative for bacterial or fungal etiology. Bronchoalveolar lavage confirmed evidence of PCP as well as CMV by PCR with no evidence of fungal elements. His blood CMV viral titers were over 3.9 × 10^6 ^copies/ml. The child succumbed to these complications in spite of aggressive therapy including ganciclovir, trimethoprim sulfisoxazole, pentamidine, broad spectrum antibacterial as well as antifungal therapies. The family opted against postmortem examination.

## Pathologic findings

### Morphologic analysis

**Figure 1:**
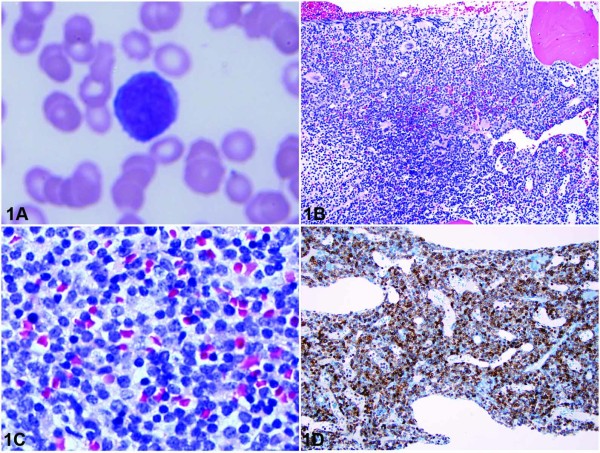
**A, Peripheral blood smear showing a lymphoma cell with relatively condensed chromatin and prominent nucleolus**. (Wright-Giemsa, original magnification ×1000). B, Bone marrow core biopsy showing hypercellular marrow with complete replacement by a diffuse lymphocytic infiltrate (Hematoxylin-Eosin, original magnification ×200). C, Bone marrow core biopsy showing large lymphocytes with relatively condensed chromatin and occasional nucleoli (Hematoxylin-Eosin, original magnification ×400). D, CD117 immunohistochemistry of bone marrow core biopsy showing strong membranous and cytoplasmic staining of tumor cells (original magnification ×200). Please refer to subsection entitled "Morphologic Analysis" for further details.

The hemogram revealed anemia normal leukocyte count (10.8 × 10^3^/μl; reference range, 4.8-10.8 × 10^3^/mL) with neutropenia (ANC 648/μl reference range 1,500-7,600) and lymphocytosis (94%; reference range, 20%-40%). The Wright-stained peripheral blood smear revealed leukemic cells having high N/C ratio, relatively dense chromatin and occasional prominent nucleoli (Figure 1A). The Wright-stained bone marrow aspirate smears showed a population of small to medium sized lymphoid cells comprising all nucleated cells. These cells were similar in morphology to those seen in the peripheral blood. The hematoxylin-eosin-stained bone marrow biopsy was highly cellular (90-95%) and displayed sheets of immature lymphoid cells (Figure 1B and 1C)

### Immunophenotypic analysis

**Figure 2:**
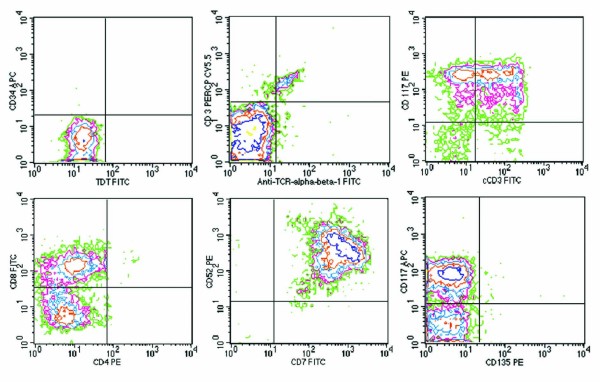
**Flow cytometry dot plots showing lymphoma cells positive for cytoplasmic CD3 (cCD3), CD8, CD117, CD7, CD52, and negative for surface CD3, CD4, TCR alpha/beta, TdT, CD34, and CD135**. Please refer to subsection entitled "Immunophenotypic Analysis" for further details.

Immunohistochemistry was performed on paraffin-embedded sections of the bone marrow core biopsy. Antibodies directed against CD3 and CD117 strongly reacted to tumor cells (Figure 1D), and an antibody directed CD20 showed no reaction. The CD3 and CD20 antibodies were purchased from Ventana Medical Systems^® ^(Oro Valley, AZ) and the CD117 antibody was purchased from Cell Marque Corporation^© ^(Rocklin, CA). Flow cytometric analysis was performed on bone marrow aspirate using a FACSCalibur™ 2000 flow cytometer and monoclonal antibodies purchased from Becton Dickinson (San Jose, CA). Flow cytometry showed a marked expansion of T cells (95% of the total lymphocyte gate) that were moderately positive for CD5, CD8, CD38, cytoplasmic CD3, CD117, and strongly positive for CD45, CD7 and CD52. The expanded T-cell population was negative for CD2, surface CD3, surface TCR-alpha/beta, surface TCR-gamma/delta, CD4, CD56, CD57, CD10, CD34, CD1a, TdT, and CD135 (Figure 2).

### Cytogenetic analysis

**Figure 3:**
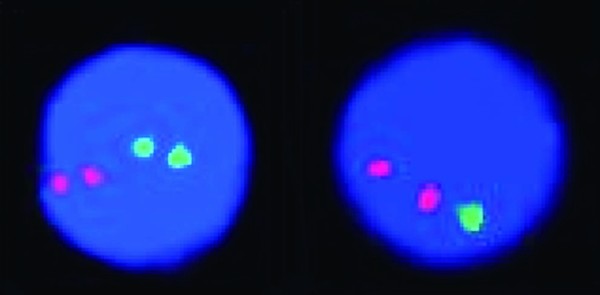
**Interphase FISH using the LSI TEL/AML1 ES (12p13 green/21q22 orange) probe, showing a normal signal pattern of two orange and two green (2O2G) on the left and the abnormal signal pattern of two orange and one green (2O1G) on the right, indicating a deletion of 12(p13)**. Please refer to subsection entitled "Cytogenetic Analysis" for further details.

G-banded metaphase analysis and fluorescence in situ hybridization (FISH) were performed on bone marrow aspirate using standard cytogenetic techniques. Fourteen trypsin-Giemsa banded metaphases from a 24 hour culture were analyzed revealing a normal male karyotype (46, XY). FISH was performed with the CEP 4 (4p11-q11), LSI BCR/ABL DC DF (9q34,22q11.2), CEP 10 (10p11-q11.1), LSI MLL DC BAR (11q23), LSI ATM (11q22.3), CEP 11 (11p11.11-q11), LSI TEL/AML1 ES (12p13/21q22), CEP 17 (17p11.1-q11.1 (D17Z1)), LSI MYC DC BAR (8q24), LSI IGH DC BAR (14q32), CEP 8 (8p11.1 - q11.1(D8Z2)) and LSI TCR alpha/delta DC BAR (14q11.2) probes, and analyzed on 200 interphase nuclei per probe. All probes were purchased from Abbott Molecular^© ^(Des Plaines, IL). A deletion of 12 (p13) was demonstrated in 97% of the nuclei analyzed (Figure 3). All other probes displayed a normal signal pattern.

### Molecular analysis

**Figure 4:**
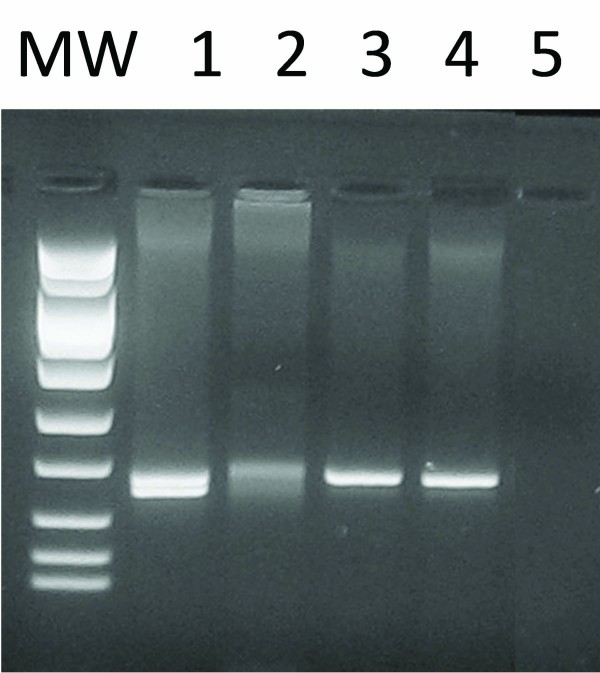
**Clonality analysis of T-cell receptor gamma chain gene by PCR showing a discrete band indicative of a monoclonal rearrangement**. Lane 1, positive control. Lane 2, normal control. Lane 3 and 4, patient sample. Lane 5, blank. MW, molecular weight marker. Please refer to subsection entitled "Molecular Analysis" for further details.

DNA was extracted from formalin-fixed, paraffin-embedded (FFPE) tissue sections of bone marrow using the QIAamp DNA FFPE Tissue kit (QIAGEN Inc., Valencia, CA). Polymerase chain reaction (PCR) amplification was performed using Techne Thermal Cycler TC3000 (Techne Inc. Burlington, NJ). The PCR reaction mix contained Taq polymerase, MgCl_2_, and 10 × buffer (AmpliTaq Gold, Roche Biochemicals, CA), dNTPs (GeneAmp^® ^dNTP MIX., Roche Biochemicals, CA) and primers for the variable, V and joining, J regions of the TCR-G (T-cell receptor gamma) chain. The amplification products were subsequently analyzed by electrophoresis on a 3% MetaPhor agarose gel (Lonza Rockland, Inc., Rockland, ME). In this analysis a discrete 170 bp band indicated the presence of a monoclonal T-cell population as previously described [5-7] (Figure 4).

## Discussion of case and review of literature

T-PLL is a rare disease, representing approximately 2% of all mature lymphocytic leukemias in adults [8] and 3% of T-cell malignancies overall [9]. T-PLL was initially described in a patient who presented with clinical and morphologic features similar to B-PLL, but in whom the cells were shown to be E-rosette positive, indicating a T-cell phenotype [10]. Following a series of studies by Matutes and coworkers between 1986 and 1991, T-PLL became established as a distinct T-cell malignancy [8,11,12].

The most common symptoms at presentation are splenomegaly (73%), followed by lymphadenopathy (53%), hepatomegaly (40%), and cutaneous lesions such as maculopapular rash and focal erythroderma (27%). Serous effusions, predominantly pleural and pericardial, are present in about 15% of patients at presentation and in up to 37% of patients later in the course of the disease [13]. Seventy-five percent of these patients had an elevated white blood cell count (typically > 100 k/mcl) at presentation. T-PLL has a distinctively short prodromal period, with about 63% of patients reporting leukemia-related symptoms for a median of two months prior to diagnosis. This is in contrast with other mature T-cell malignancies such as Sézary Syndrome and T-LGL, which have prodromes with a medium duration of 24-38 months and 9 months, respectively [13].

Marked morphologic heterogeneity has been observed in cases of T-PLL. Over three quarters of cases show typical prolymphocyte morphology that may be indistinguishable from that of B-PLL, namely medium sized lymphoid cells with moderately condensed nuclear chromatin and prominent central nucleoli. T-prolymphocytes tend to have more intense cytoplasmic basophilia and nuclear irregularity than B-prolymphocytes. In some cases, cytoplasmic protrusions or blebs may be present. In about 20% of T-PLL cases, the cells are smaller with more condensed nuclear chromatin and inconspicuous nucleoli. Such cases were originally referred to as T-CLL, but now are classified as the small cell variant of T-PLL. Less commonly, in about 5% of cases, the cells show markedly irregular nuclei resembling those seen in Sézary cells. While originally labeled as Sézary cell leukemia, these cases are now designated as the cerebriform variant of T-PLL. Both variants were reclassified because they shared the same immunophenotypic and cytogenetic features as typical cases of T-PLL [14].

The immunophenotype of T-prolymphocytes is consistent with that of a mature post-thymic T-cell. They are negative for CD1a and TdT, but positive for pan T-cell markers such as CD2, CD3, CD5, and CD7. CD7 is expressed with stronger intensity than seen in normal T-cells and other mature T-cell malignancies, but at levels comparable to those seen in T-ALL [15]. Surface expression of CD3 and TCR-α/β may not be detected in up to 20% of cases, but their expression is always seen in the cytoplasm [15]. While most cases (65%) consist of CD4+/CD8- cells, 21% of cases are CD4+/CD8+ and 13% of cases are CD4-/CD8+ [8]. There is variable expression of antigens related to T-cell activation, such as CD25, CD38, and HLA-DR. While not a lymphocyte specific marker, CD52 is more strongly expressed in T-prolymphocytes than in normal T-cells [16].

While the immunophenotype of our case is predominantly that of a mature T-cell malignancy, the expression of CD117 (c-kit) seems counterintuitive. CD117 is normally expressed by a subset of thymocytes during normal lymphopoiesis which have not yet undergone rearrangement of their T-cell receptor (TCR) genes [17], playing a vital role in early T-cell development. This marker is also normally expressed by hematopoietic progenitor cells of all lineages [17]. Therefore CD117 serves as a surrogate marker of immaturity, with expression in a subset of precursor T-cell lymphoblastic neoplasms [18,19]. A review of the literature for the incidence of CD117 expression in mature T-cell malignancies showed CD117 expression restricted to CD4-/CD8+ cases [20], consistent with the present case.

Many cytogenetic studies have identified several recurrent chromosomal abnormalities in T-PLL. Up to 90% of patients have abnormalities of chromosome 14 that can be demonstrated by FISH. The majority of these abnormalities consist of either inv(14)(q11q32) or t(14;14)(q11;q32) [1]. Inv(14)(q11q32) alone can be detected by conventional cytogenetic studies in more than two-thirds of cases [21]. About 19% of patients have karyotypes showing abnormalities of Xq28, the most common of which is t(X;14)(q28;q11) [1]. Such rearrangements are now considered to be a genetic hallmark of T-PLL and a primary event in its oncogenesis. Both inv(14)(q11q32) or t(14;14)(q11;q32) result in the overexpression of the TCL1 oncogene located on 14q32.1 through its juxtaposition to the TCR-alpha gene on 14q11. When overexpressed, the TCL1 protein binds the D3 phosphoinositide-regulated kinase AKT1 in the cytoplasm, augmenting its transport into the nucleus. This results in increased expression of genes responsible for promoting T-cell proliferation and survival [22]. Likewise, t(X;14)(q28;q11) juxtaposes the MCTP1 (mature T-cell proliferation 1) gene to TCR-alpha gene. MCTP1 is a homologue of TCL1 that also binds to AKT1 [23,24]. Interestingly, inv(14)(q11q32), t(14;14)(q11;q32), and t(X;14)(q28;q11) have all been detected in expanding T-cell clones from patients with ataxia-telangiectasia. Such clones are usually subclinical and identified as incidental findings several years before lymphoma develops [3].

Besides the aberrations on chromosome 14, most T-PLL cells usually harbor other secondary chromosomal abnormalities. Unbalanced rearrangements of chromosome 8 are the most common secondary abnormalities and have been reported in up to 80% of cases. Such chromosome 8 abnormalities include trisomy 8q, isochromosome 8(q10), t(8;8)(p12;q11), and translocations with other chromosomes [25]. In addition, c-myc protein amplification has been demonstrated by flow cytometry [26]. Despite their high frequency, the role of these chromosome 8 abnormalities in the pathogenesis of T-PLL remains to be elucidated.

Although conventional cytogenetics revealed a normal karyotype, a comprehensive panel of FISH probes detected an isolated deletion of the (p13) region of chromosome 12. As a result of limited resolution, this deletion cannot be visualized using standard cytogenetic techniques (i.e. karyotyping) and requires FISH for detection. It is present in nearly half of T-PLL cases [27], but has always been accompanied by the well characterized aberrations of either chromosomes 14, X, or 8 [1,12,28,29]. Considering that abnormalities of 14q11 [i.e. t(14;14)(q11;q32), inv(14)(q11q32), t(X;14)(q28;q11)] and chromosome 8 [i.e. i(8)(q10), t(8;8)(p12;q11)] are each present in up to 90% and 80% of T-PLL patients, respectively [1,23], we believe that the present case is the first report of a patient with an isolated deletion of the 12(p13) region. A review of the literature for T-PLL cases lacking the common abnormalities of chromosomes 14, X, and 8 showed an overrepresentation of CD4-/CD8+ T-PLL cases like ours [12,30]. Therefore our case may represent a subset of T-PLL cases in which alternative pathways are likely responsible for its pathogenesis. The minimal region of 12(p13) deletion contains the CDKN1B gene, which encodes the cell cycle regulatory protein p27KIP1 [27,31]. Decreased expression of this protein results in stabilization of cyclinD-CDK4/6 complexes and facilitates cell cycle progression [32], which was shown to be sufficient for the development of T-PLL in mouse models [31].

The most important differential diagnosis in the pediatric age group is T-ALL. T-ALL typically presents as a mediastinal mass with an immunophenotype consistent with late cortical thymocytes (i.e. positive for TdT, CD1a, cytoplasmic CD3, CD4, and CD8). T-ALL cells may range from small, round cells with high N/C ratios, relatively condensed chromatin and inconspicuous nucleoli to larger cells with abundant basophilic cytoplasm, irregular nuclear contour, dispersed chromatin and one or more distinct nucleoli [14]. It is this cytologic heterogeneity that may pose a challenge in differentiating T-ALL from T-PLL in a child, since it may overlap with the T-PLL cytologic spectrum. Furthermore, as previously mentioned, rare cases of T-PLL will aberrantly express CD117, a marker more commonly seen in up to 11% of T-ALL cases. Importantly, T-ALL cases that express CD117 also carry activating mutations of the FLT3 receptor tyrosine kinase (i.e., CD135), the genetic abnormality most commonly seen in AML [18]. In contrast, our case did not express CD135 by flow cytometry and no mutations were detected by PCR (data not shown), arguing against a diagnosis of T-ALL.

The WHO categorizes T-cell and NK-cell neoplasms into broad clinically defined groups such as leukemic or disseminated, nodal, extranodal, and cutaneous [14]. The most common mature T-cell malignancy in children is anaplastic lymphoma kinase (ALK) positive anaplastic large cell lymphoma (ALCL), account for up to 30% of lymphomas in the pediatric age group. It has been suggested that a majority of non-ALCL T-cell/NL-cell lymphomas in children tend to be derived from components of the innate immune system such as cytotoxic T or NK cells [33]. Consistent with this, CD4-/CD8+ T-cell lymphomas were observed in young transgenic mice models of T-PLL [34]. Therefore it appears that evolutionary primitive components of the immune system are featured more often in pediatric hematologic malignant neoplasms, and conversely tumors of the adaptive immune system, a more mature component of the immune system, are exceptionally rare in children [33]. The frequency of non-anaplastic mature T-cell lymphomas in children is too low for performing large scale clinical trials. Therefore not much is known about their prognosis and optimal choice of therapy.

Standard T-cell ALL induction therapy was not effective in this case. FLACC regimen was effective in achieving remission status while definitive therapy is most likely with stem cell transplantation. This case illustrates that the diagnosis of T-PLL should be entertained in all children with T-ALL and alternative induction regimens such as the one used in this case should be entertained early in the course of treatment.

FLACC regimen is highly immunosuppressive. Reactivation of CMV should be considered in all cases where evidence of prior CMV exposure is present. This may happen in spite of standard prophylactic use of valacyclovir, and periodic monitoring of peripheral blood CMV viral load may be appropriate. Those children with no evidence of prior CMV exposure should receive CMV-negative blood products and screened periodically for new exposure. Similarly, although pentamidine was used to prevent PCP pneumonitis, it was ineffective in this case. Trimethoprim/sulfisoxazole, although may lead to prolonged neutropenia, may be more effective and should be considered.

To our knowledge, the present case is the first report of T-PLL in a child. The lack of underlying ataxia-telangiectasia makes this a case of sporadic T-PLL, which is even more intriguing. While T-ALL is the more commonly seen T-cell malignancy in the pediatric age group, this case highlights the importance of keeping mature T-cell malignancies in the differential diagnosis. Furthermore, the regimen successfully used in this case may provide a valuable option for clinicians attempting to treat rare cases of pediatric mature T-cell malignancies. The isolated deletion 12(p13) in our case highlights the importance of alternative mechanisms of T-PLL leukemogenesis that needs to be further investigated. Finally, additional studies are necessary to determine the prognostic significance of CD117 expression in mature T-cell malignancies and its restriction to CD4-/CD8+ cases.

## Consent

Written informed consent was not obtained because the patient is deceased and no next of kin are available.

## Competing interests

The authors declare that they have no competing interests.

## Authors' contributions

MB searched the literature and drafted the manuscript. AS helped draft the manuscript. AZ supplied the cytogenetics data and helped draft the cytogenetics section of manuscript. SS supplied the molecular data and helped draft the molecular section manuscript. MG supplied the flow cytometry data. MC clinically managed the patient, supplied clinical data, and helped draft the clinical sections of the manuscript. YH helped draft the manuscript. YM supplied the morphologic images and helped draft the manuscript. TA helped draft the manuscript. All authors read and approved the final manuscript.

## References

[B1] Maljaei SH Brito-Babapulle V Hiorns LR Catovsky D Abnormalities of chromosomes 8, 11, 14, and X in T-prolymphocytic leukemia studied by fluorescence in situ hybridization Cancer Genet Cytogenet 1998 103 110 116 10.1016/S0165-4608(97)00410-X 9614908

[B2] Melo JV Catovsky D Galton DA The relationship between chronic lymphocytic leukaemia and prolymphocytic leukaemia. I. Clinical and laboratory features of 300 patients and characterization of an intermediate group Br J Haematol 1986 63 377 387 3487341

[B3] Taylor AM Metcalfe JA Thick J Mak YF Leukemia and lymphoma in ataxia telangiectasia Blood 1996 87 423 438 8555463

[B4] Williams KM Higman MA Chen AR Successful treatment of a child with late-onset T-cell post-transplant lymphoproliferative disorder/lymphoma Pediatr Blood Cancer 2008 50 667 670 10.1002/pbc.21171 17318876

[B5] Benhattar J Delacretaz F Martin P Chaubert P Costa J Improved polymerase chain reaction detection of clonal T-cell lymphoid neoplasms Diagn Mol Pathol 1995 4 108 112 10.1097/00019606-199506000-00006 7551290

[B6] Beltran B Castillo J Salas R Quinones P Morales D Hurtado F ALK-positive diffuse large B-cell lymphoma: report of four cases and review of the literature J Hematol Oncol 2009 2 11 10.1186/1756-8722-2-11 19250532 PMC2651189

[B7] Wang L Zhu K Zha X Chen S Yang L Chen S Li Y Evolution of T-cell clonality in a patient with Ph-negative acute lymphocytic leukemia occurring after interferon and imatinib therapy for Ph-positive chronic myeloid leukemia J Hematol Oncol 2010 3 14 10.1186/1756-8722-3-14 20377918 PMC2859394

[B8] Matutes E Brito-Babapulle V Swansbury J Clinical and laboratory features of 78 cases of T-prolymphocytic leukemia Blood 1991 78 3269 3274 1742486

[B9] Bartlett NL Longo DL T-small lymphocyte disorders Semin Hematol 1999 36 164 170 10319385

[B10] Catovsky D Galetto J Okos A Galton DA Wiltshaw E Stathopoulos G Prolymphocytic leukaemia of B and T cell type Lancet 1973 2 232 234 4124423 10.1016/s0140-6736(73)93135-8

[B11] Matutes E Garcia Talavera J O'Brien M Catovsky D The morphological spectrum of T-prolymphocytic leukaemia Br J Haematol 1986 64 111 124 10.1111/j.1365-2141.1986.tb07579.x 3489482

[B12] Brito-Babapulle V Pomfret M Matutes E Catovsky D Cytogenetic studies on prolymphocytic leukemia. II. T cell prolymphocytic leukemia Blood 1987 70 926 931 3115337

[B13] Herling M Khoury JD Washington LT Duvic M Keating MJ Jones D A systematic approach to diagnosis of mature T-cell leukemias reveals heterogeneity among WHO categories Blood 2004 104 328 335 10.1182/blood-2004-01-0002 15044256

[B14] Jaffe ES Chaganti RSK Nanjangud G Memorial Sloan-Kettering Cancer Center (MSKCC) Hematopathology 2011 1 Philadelphia, PA: Saunders/Elsevier xiii 1058

[B15] Ginaldi L Matutes E Farahat N De Martinis M Morilla R Catovsky D Differential expression of CD3 and CD7 in T-cell malignancies: a quantitative study by flow cytometry Br J Haematol 1996 93 921 927 10.1046/j.1365-2141.1996.d01-1720.x 8703826

[B16] Ginaldi L De Martinis M Matutes E Levels of expression of CD52 in normal and leukemic B and T cells: correlation with in vivo therapeutic responses to Campath-1H Leuk Res 1998 22 185 191 10.1016/S0145-2126(97)00158-6 9593475

[B17] Broudy VC Stem cell factor and hematopoiesis Blood 1997 90 1345 1364 9269751

[B18] Paietta E Ferrando AA Neuberg D Activating FLT3 mutations in CD117/KIT(+) T-cell acute lymphoblastic leukemias Blood 2004 104 558 560 10.1182/blood-2004-01-0168 15044257

[B19] Sperling C Schwartz S Buchner T Thiel E Ludwig WD Expression of the stem cell factor receptor C-KIT (CD117) in acute leukemias Haematologica 1997 82 617 621 9407735

[B20] Gorczyca W Differential diagnosis of T-cell lymphoproliferative disorders by flow cytometry multicolor immunophenotyping. correlation with morphology Methods Cell Biol 2004 75 595 621 15603444 10.1016/s0091-679x(04)75025-6

[B21] Dungarwalla M Matutes E Dearden CE Prolymphocytic leukaemia of B- and T-cell subtype: a state-of-the-art paper Eur J Haematol 2008 80 469 476 10.1111/j.1600-0609.2008.01069.x 18331594

[B22] Pekarsky Y Koval A Hallas C Tcl1 enhances Akt kinase activity and mediates its nuclear translocation Proc Natl Acad Sci USA 2000 97 3028 3033 10.1073/pnas.040557697 10716693 PMC16186

[B23] Stern MH Soulier J Rosenzwajg M MTCP-1: a novel gene on the human chromosome Xq28 translocated to the T cell receptor alpha/delta locus in mature T cell proliferations Oncogene 1993 8 2475 2483 8361760

[B24] Pekarsky Y Hallas C Isobe M Russo G Croce CM Abnormalities at 14q32.1 in T cell malignancies involve two oncogenes Proc Natl Acad Sci USA 1999 96 2949 2951 10.1073/pnas.96.6.2949 10077617 PMC15875

[B25] Mossafa H Brizard A Huret JL Trisomy 8q due to i(8q) or der(8) t(8;8) is a frequent lesion in T-prolymphocytic leukaemia: four new cases and a review of the literature Br J Haematol 1994 86 780 785 10.1111/j.1365-2141.1994.tb04829.x 7918072

[B26] Maljaie SH Brito-Babapulle V Matutes E Hiorns LR De Schouwer PJ Catovsky D Expression of c-myc oncoprotein in chronic T cell leukemias Leukemia 1995 9 1694 1699 7564512

[B27] Hetet G Dastot H Baens M Recurrent molecular deletion of the 12p13 region, centromeric to ETV6/TEL, in T-cell prolymphocytic leukemia Hematol J 2000 1 42 47 10.1038/sj.thj.6200008 11920168

[B28] Soulier J Pierron G Vecchione D A complex pattern of recurrent chromosomal losses and gains in T-cell prolymphocytic leukemia Genes Chromosomes Cancer 2001 31 248 254 10.1002/gcc.1141 11391795

[B29] Costa D Queralt R Aymerich M High levels of chromosomal imbalances in typical and small-cell variants of T-cell prolymphocytic leukemia Cancer Genet Cytogenet 2003 147 36 43 10.1016/S0165-4608(03)00161-4 14580769

[B30] Ascani S Leoni P Fraternali Orcioni G T-cell prolymphocytic leukaemia: does the expression of CD8+ phenotype justify the identification of a new subtype? Description of two cases and review of the literature Ann Oncol 1999 10 649 653 10.1023/A:1008349422735 10442186

[B31] Le Toriellec E Despouy G Pierron G Haploinsufficiency of CDKN1B contributes to leukemogenesis in T-cell prolymphocytic leukemia Blood 2008 111 2321 2328 10.1182/blood-2007-06-095570 18073348

[B32] Sherr CJ Roberts JM Living with or without cyclins and cyclin-dependent kinases Genes Dev 2004 18 2699 2711 10.1101/gad.1256504 15545627

[B33] Jaffe ES Pathobiology of peripheral T-cell lymphomas Hematology Am Soc Hematol Educ Program 2006 1 317 322 17124078 10.1182/asheducation-2006.1.317

[B34] Stern MH Transgenic models of T-cell prolymphocytic leukaemia Haematologica 1999 84 Suppl EHA-4 64 66 10907471

